# Supplementary material for: Optimizing the Integration of Microwave Processing and Enzymatic Extraction to Produce Polyphenol-Rich Extracts from Olive Pomace
Source: Foods. 2023 Oct 12;12(20):3754. doi: 10.3390/foods12203754 (PMC10606511; doi:10.3390/foods12203754)
Supplement: Supplementary file 1 [file foods-12-03754-s001.zip › foods-2617067-supplementary.pdf]

Optimization of combined microwave- and enzyme-assisted green extraction aiming to produce polyphenols-rich extracts from olive pomace

Gabriela A. Macedo[1,2]; Paula de P. M. Barbosa[1*]; Fernanda F. G. Dias[2], Juliana M. L. N. M. Bell[2,3]

[1]Bioprocesses Laboratory. DEPAN/FEA (School of Food Engineering)/Unicamp (University of Campinas), R. Monteiro Lobato, 80, 13083970, Campinas, Brazil

[2]Department of Food Science and Technology, University of California, Davis, One Shields Avenue, Davis, CA 95616, United States

[3]Biological and Agricultural Engineering, University of California, Davis, One Shields Avenue, Davis, CA, 95616, United States

[*]Corresponding author: paulamenezesbarbosa@gmail.com

1. Supplementary material

1.1. Tables

Table S1. Statistical analysis of the results of the 2^{4-1} FFD used to determine the significant parameters for MEAE-Tan.

	Effect	Std. error	*P-value*
Mean/Intercept	308.77	15.14	0.000001
X1 (Temperature)	7.48	30.38	0.400612
X2 (Ramp and hold time)	15.81	30.38	0.621496
X3 Pomace:water ratio	-37.73	30.38	0.260623
Enzyme concentration	215.93	30.38	0.000390

Table S2. Statistical analysis of the results of the 2^{4-1} FFD used to determine the significant parameters for MEAE-Tan-Cel-Pec.

	Effect	Std. error	*P-value*
Mean/Intercept	112.95	3.12	<0.000001
X1 (Temperature)	32.56	6.25	0.002001
X2 (Ramp and hold time)	-10.93	6.25	0.130885
X3 Pomace:water ratio	14.56	6.25	0.058748
Enzyme concentration	47.06	6.25	0.000285

Table S3. Analysis of variance (ANOVA) of MEAE-Tan results in 15 min of reaction.

Source of variation	SS	DF[a]	MS	Fcal	Ftab	F Test[b]
Regression	21179609.4	5	4235921.886	16.43	5.05	3.254
Residue	1288846.3	5	257769.2686			
Lack of fit	1225659.3	3	408553.0871	12.93	19.16	0.675
Pure error	63187.1	2	31593.54093			
Total	22468455.8	10				

[a]Degree of freedom. [b]F (0.95; 5.5) = 5.05; F (0.95; 3.2) = 19.16. R^2 = 0.94264.

Table S4. Role of enzyme concentration and pomace/water ratio on the extractability of OP phenolics using MEAE-Tan in 15 min of reaction (2^2 CCRD results at 15 min and validation of the predictive model).

Run	Variables		Total phenolic content (mg GAE/kg pomace)		
	X1 Enzyme concentration (%, w/w)	X2 Pomace:water ratio (w/w)	Yexp[a]	Ypred[b]	ΔY[c]
1	(−1) 0.9	(−1) 1:11	3611.3	3835.3	224.0
2	(1) 2.1	(−1) 1:11	5053.86	5661.6	607.8
3	(−1) 0.9	(1) 1:4.7	1768.19	1333.7	−434.5
4	(1) 2.1	(1) 1.4.7	3440.32	3389.6	−50.7
5	(−1.41) 0.66	(0) 1:6.6	1397.06	1582.1	185.1
6	(+1.41) 2.34	(0) 1:6.6	4678.48	4319.2	−359.3
7	(0) 1.5	(−1.41) 1:15	6388.8	5834.7	−554.1
8	(0) 1.5	(+1.41) 1:4	2089.38	2469.3	379.9
9	(0) 1.5	(0) 1:6.6	2723.54	2929.1	205.6
10	(0) 1.5	(0) 1:6.6	3020.08	2929.1	−91.0
11	(0) 1.5	(0) 1:6.6	3041.6	2929.1	−112.5
**Validation**	**(2.34) 1.41**	**(1:15) −1.41**	**7583.11**	**7110.6**	**−472.5**

[a]Experimental value. [b]Value predicted by the quadratic model. [c]Difference between predicted and experimental values.

Table S5. Role of temperature, enzyme concentration, and pomace/water ratio on the extractability of OP phenolics using MEAE-Tan-Cel-Pec in 15 min of reaction (2^3 CCRD results at 15 min and validation of the predictive model).

Run	Variables			Total phenolic content (mg GAE/kg pomace)		
	X1 Temperature (°C)	X2 Enzyme concentration (%, w/w)	X3 Pomace:water ratio (w/w)	Ypred[a]	Yexp[b]	ΔY[c]
1	(−1) 55	(−1) 0.9	(−1) 1:11	1403.45	1201.58	201.9
2	(1) 65	(−1) 0.9	(−1) 1:11	1226.62	1037.43	189.2
3	(−1) 55	(1) 2.1	(−1) 1:11	1944.94	1874.3	70.6
4	(1) 65	(1) 2.1	(−1) 1:11	1768.32	1591.75	176.6
5	(−1) 55	(−1) 0.9	(1) 1:4.7	657.74	720.3	−62.6
6	(1) 65	(−1) 0.9	(1) 1:4.7	610.59	567.22	43.4
7	(−1) 55	(1) 2.1	(1) 1:4.7	728.51	803.69	−75.2
8	(1) 65	(1) 2.1	(1) 1:4.7	681.56	769,42	−87.9
9	(−1.68) 46.5	(0) 1.5	(0) 1:6.6	995.61	1020.9	−25.3
10	(+1.68) 73.5	(0) 1.5	(0) 1:6.6	807.63	943.92	−136.3
11	(0) 60	(−1.68) 0.5	(0) 1:6.6	527.30	693.72	−166.4
12	(0) 60	(+1.68) 2.0	(0) 1:6.6	1041.77	1036.94	4.8
13	(0) 60	(0) 1.5	(−1.68) 1:20	2390.02	2715.01	−325.0
14	(0) 60	(0) 1.5	(+1.68) 1:4	850.75	687.34	163.4
15	(0) 60	(0) 1.5	(0) 1:6.6	696.33	629.56	66.8
16	(0) 60	(0) 1.5	(0) 1:6.6	696.33	735.42	−39.1
17	(0) 60	(0) 1.5	(0) 1:6.6	696.33	695.32	1.0
**Validation**	**(−1.68) 46.5**	**(1.68) 2.0**	**(−1.68) 1:20**	**2793.00**	**2938.25**	**−145.25**

[a]Total phenolic content predicted by the quadratic model. [b]Experimental total phenolic content. [c]Difference between predicted and experimental values.

Table S6. Analysis of variance (ANOVA) of MEAE-Tan-Cel-Pec results in 15 min of reaction.

Source of variation	SS	DF[a]	MS	Fcal	Ftab	F-Test[b]
Regression	4603728.97	9	511525.4409	11.29	3.68	3.07
Residue	317119.5	7	45302.79014			
Lack of fit	311406.6	5	62281.32438	21.80	19.3	1.13
Pure error	5712.9	2	2856.454533			
Total	4920848.5	16				

[a]Degree of freedom. [b]F (0.95; 9,7) = 3.68; F (0.95; 5.2) = 19. R^2 = 0.93.

1.2. Figures

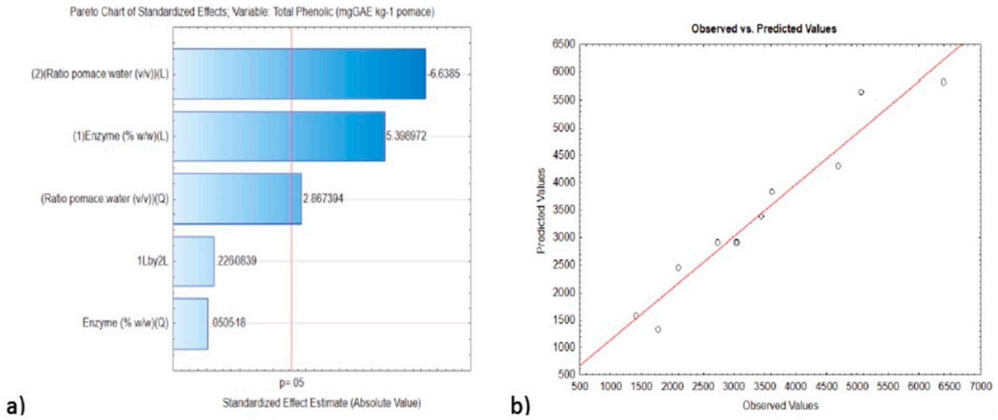

Figure S1. a) Pareto chart of standardized effects. b) Predicted versus observed total phenolic content obtained by MEAE-Tan in 15 min of reaction.

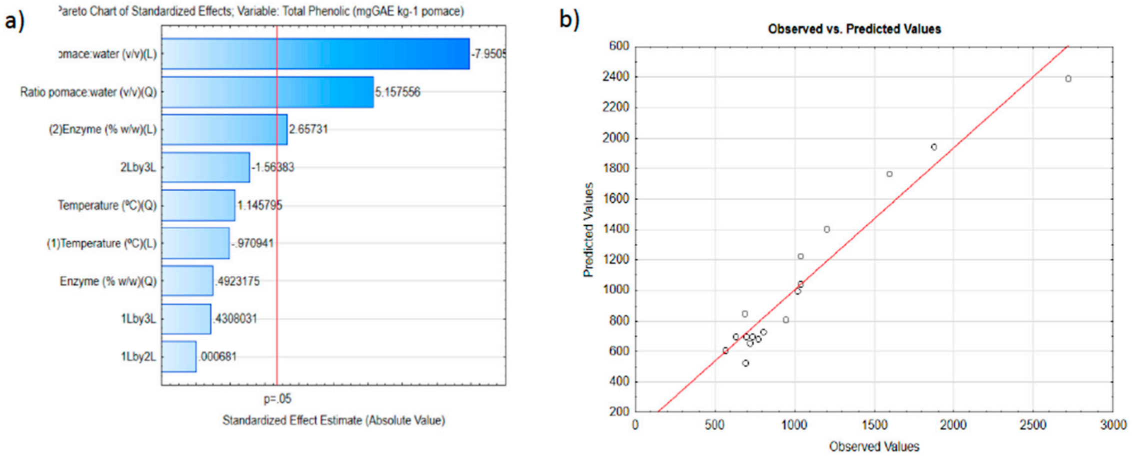

Figure S2. a) Pareto chart of standardized effects. b) Predicted versus observed total phenolic content obtained by MEAE-Tan-Cel-Pec in 15 min of reaction.
